# Bilateral Xp11.2 translocation renal cell carcinoma: a case report

**DOI:** 10.1186/s12894-018-0419-3

**Published:** 2018-11-20

**Authors:** Takashi Karashima, Takahira Kuno, Naoto Kuroda, Hirofumi Satake, Satoshi Fukata, Masakazu Chikazawa, Chiaki Kawada, Ichiro Yamasaki, Taro Shuin, Makoto Hiroi, Keiji Inoue

**Affiliations:** 1 Department of Urology, Kochi University, Kochi Medical School, Kohasu, Oko, Nankoku, Kochi 783-8505 Japan; 2 Department of Diagnostic Pathology, Kochi Red Cross Hospital, Kochi-Shi, Kochi 780-0062 Japan; 3 Department of Urology, Izumino Hospital, Kochi-Shi, Kochi 781-0011 Japan; 4 Laboratory of Diagnostic Pathology, Kochi Medical School Hospital, Kohasu, Oko, Nankoku, Kochi, 783-8505 Japan

**Keywords:** Renal cell carcinoma, Xp11.2 translocation, Bilateral, ASPL-TFE3

## Abstract

**Background:**

Xp11.2 translocation renal cell carcinoma (RCC) is a rare variety of a kidney neoplasm. We report a case of bilateral Xp11.2 translocation RCC occurring metachronously and discuss this very rare entity with reference to the literature.

**Case presentation:**

The patient was a 56-year-old woman who presented with a right renal tumor. The patient had undergone left radical nephrectomy 7 years previously, which resulted in a histopathological diagnosis of clear cell RCC. Open right partial nephrectomy was performed under the presumptive diagnosis of recurrence of clear cell RCC. The present right renal tumor was pathologically diagnosed Xp11.2 translocation RCC. More than 70% of the tumor cells in the present right tumor were strongly positive for transcription factor E3 (TFE3) expression by immunohistochemical analysis with an anti-TFE3 antibody. A break-apart of the TFE3 genes in the bilateral tumors was identified by fluorescence in situ hybridization analysis. Real time-polymerase chain reaction analysis for the alveolar soft part sarcoma locus-TFE3 fusion gene was performed, which gave a positive result in the bilateral tumors. Pathological comparison of each of the tumors might lead to a final diagnosis of Xp11.2 translocation RCC occurring metachronously.

**Conclusions:**

We present the bilateral Xp11.2 translocation RCC. A combination of immunohistochemical, cytogenetic and molecular biological approaches allowed the final diagnosis of such a rare RCC.

## Background

Xp11.2 translocation renal cell carcinoma (RCC) is a rare variety of kidney neoplasm that represents approximately 1% of RCC [1]. It is a clinically identified malignant neoplasm of kidney with an advanced stage and a poorer prognosis than conventional clear cell RCC [2]. Xp11.2 translocation RCC results from gene fusions between the transcription factor E3 (TFE3) gene located on chromosome Xp11.2 and various fusion partners. These chimeric gene fusions result in overexpression of fusion proteins that contain the C-terminal portion of TFE3. The TFE3 fusion partner genes have been recently well characterized. A common fusion partner gene is alveolar soft part sarcoma critical region 1 (ASPSCR1), der(17)t(X;17)(p11.2;q25). This unbalanced translocation results in fusion of the TFE3 gene, a member of the basic-helix-loop-helix family of transcription factors, on Xp11.2, to a novel gene named alveolar soft part sarcoma locus (ASPL) on 17q25 [3]. Other common fusion genes are papillary renal cell carcinoma-TFE3 (PRCC-TFE3), t(X;1)(p11.2;q21.2) and PTB-associated splicing factor-TFE3 (PSF-TFE3), t(X;1)(p11.2;p34) [4, 5]. Less commonly observed gene fusions are NonO-TFE3 inv.(X)(p11.2;q12) and clathrin heavy chain-TFE3 (CLTC-TFE3), (X;17)(p11.2;q23) [6, 7].

In this report, we present an extremely rare case of bilateral Xp11.2 translocation RCC occurring metachronously, and discuss the uncommon features of this case as determined by histopathological, cytogenetic and molecular approaches.

## Case presentation

**Fig. 1:**
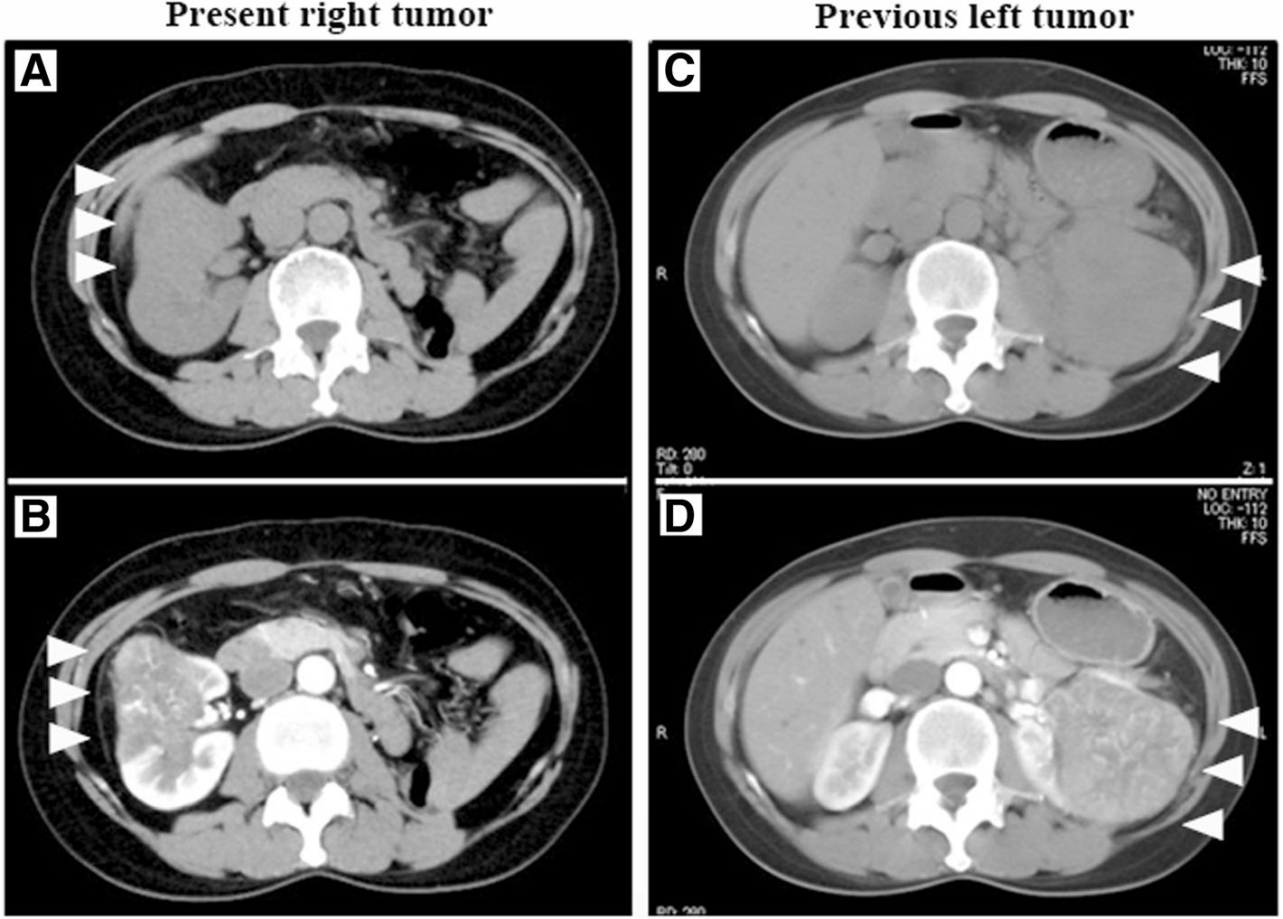
Pre-operative diagnostic imaging of the present and the previous tumor. Abdominal CT images of the present right renal tumor (**a**, **b**) and the previous left renal tumor (**c**, **d**). The present right renal tumor was 5.3 cm in diameter and showed poorly-defined margins and an irregular contrast. The previous left renal tumor was 7.0 cm in diameter, and showed well-defined margins and an irregular contrast

A 56-year-old woman was introduced to Kochi Medical School from a private hospital for right renal tumor detected by abdominal computed tomography (CT). She had been undergone radical nephrectomy for left renal cell carcinoma (RCC) 7 years before. An abdominal CT of the present tumor revealed a right renal tumor, 5.3 cm in diameter, showing poorly-defined margins, irregular contrast and no findings of metastases (Fig. 1a, b). An abdominal CT that was performed 7 years ago revealed a left renal tumor, 7.0 cm in diameter, showing well-defined margins, irregular contrast and no findings of metastases, diagnosed clinical stage T1b N0 M0 left RCC (Fig. 1c, d). She did not have any other medical history or family history.

**Fig. 2:**
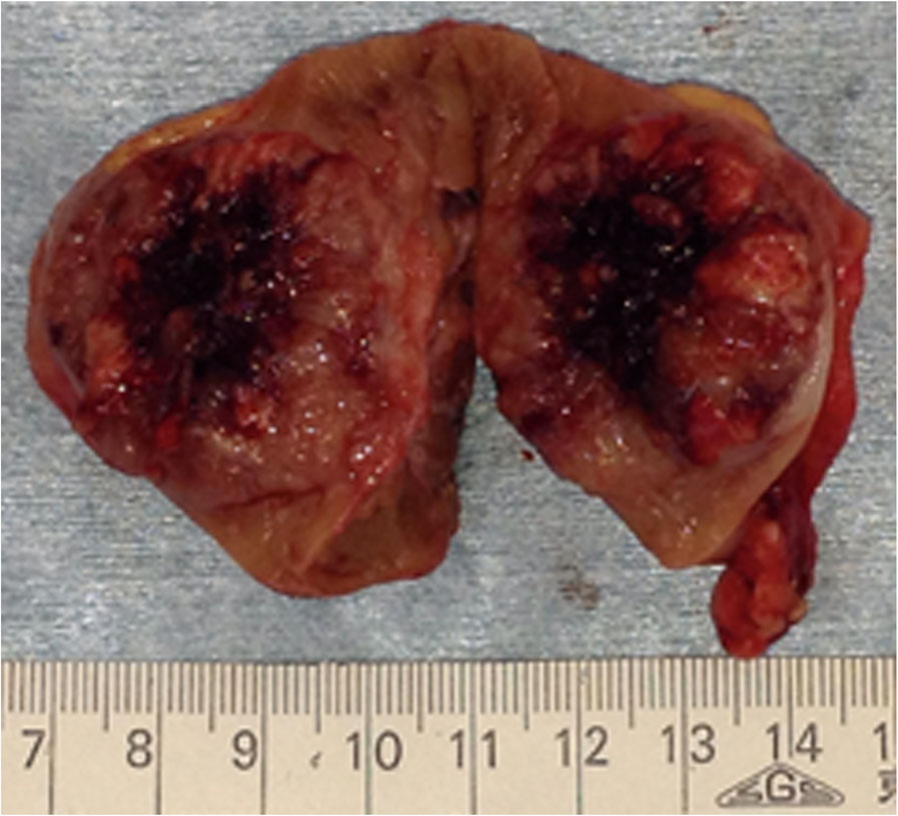
Macroscopic findings of the present right tumor. The present right tumor resected by partial nephrectomy was macroscopically a well-marginated solid mass. The cross-sectional surface was lobulated and heterogenously yellow to brown with bleeding and necrosis

**Fig. 3:**
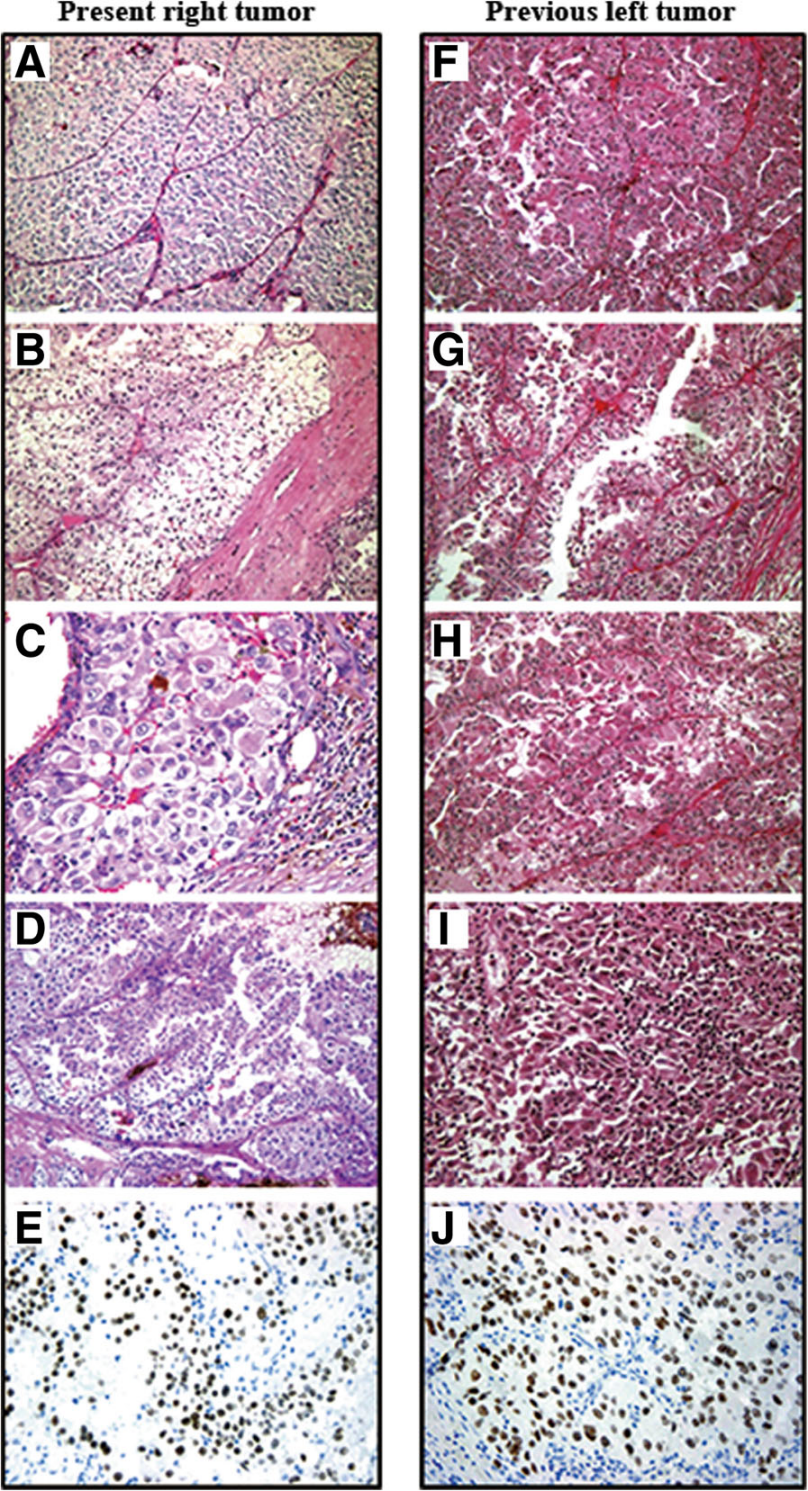
Microscopic findings of the present right tumor and previous left tumor. HE staining of the present right tumor mostly showed an alveolar growth pattern (× 100; **a**) with cells composed clear cytoplasm (× 100; **b**). Very large tumor cells (× 100; **c**) and a papillary growth pattern (× 100; **d**) were focally observed. Moderate to strong immunostaining of TFE3 in the nuclei of tumor cells was seen (× 200; **e**). HE staining of the previous left tumor showed an alveolar growth pattern (× 100; **f**), pale eosinophilic cytoplasm (× 100; **g**) and very large tumor cells (× 100; **h**). Dedifferentiated sarcomatoid features were partially observed (× 100; **i**). Moderate to strong immunostaining of TFE3 in the nuclei of tumor cells was seen (× 200; **j**)

Open right partial nephrectomy was performed under a presumed diagnosis of clinical stage T1b N0 M0 right RCC, recurrent or due to metastasis from the previous left tumor. The tumor was a macroscopically well-circumscribed solid mass. The cross-sectional surface was lobulated and heterogenously yellow to brown with bleeding and necrosis (Fig. 2). Microscopically, the tumor showed an alveolar growth pattern admixed with eosinophilic and clear cytoplasm. Papillary architecture was also focally seen. In some areas, eosinophilic coarse granules were identified in the tumor cytoplasm. Pathological stage was pT1b pN0 with negative surgical margin. Nuclear Grade corresponded to largely Fuhrman Grade 3 and partly Grade 4. Hyaline nodules and psammoma bodies were observed in the stroma. Immunohistochemically, the tumor cells showed diffuse positivity for renal cell carcinoma-maker (RCCMa, PN-15, 1: 100, Cell Marque, CA, USA) and cluster differentiation (CD)10 (56C16, prediluted, Novocastra Laboratories Ltd., Newcastle, UK) and negativity for Cathepsin K (3F9, Abcam, Tokyo, JP), Melanosome (Human melanoma black; HMB45, prediluted, DAKO, Glostrup, Denmark), Melan A (A103, 1: 100, Novocastra Laboratories Ltd., Newcastle, UK), and alpha smooth muscle actin (data not shown). Seventy percent of neoplastic cell nuclei stained positive for TFE3 (MRQ-37, prediluted, Ventana Medical Systems, Inc., Tucson, AZ), with a staining intensity of (moderate) 2+ to (strong) 3+ (Fig. 3). Staining for transcription factor EB (TFEB, polyclonal, V-17, 1: 400, Santa Cruz, Biotechnology, Inc., Dallas, TX) was generally negative (data not shown).

Hematoxylin and eosin, and immunohistochemical stains from the previous tumor were retrospectively reviewed. In H and E staining, tubular, papillary, and alveolar growth patterns were noted admixed with eosinophilic and clear cytoplasm. Additionally, very large tumor cells were seen and dedifferentiation with a discohesive area and rhabdoid features was also noted. Necrosis and hemorrhage were present. Pathological stage was pT1b pN0. Nuclear Grade corresponded to Fuhrman Grade 4. Small venous invasion by carcinoma cells was seen. Neoplastic cells showed diffuse immunohistochemical expression of RCCMa, CD10, Alpha-Methylacyl-CoA Race (AMACR; P504S, 13H4, 1: 100, DAKO, Glostrup, Denmark) and negative results for cytokeratin 7, Carbonic Anhydrase IX (CA9, D47G3, Cell Signaling, MA, USA), HMB45, Melan A and Cathepsin K (data not shown). TFE3 was positively stained in the nuclei of 5% of neoplastic cells with a staining intensity of 2+ to 3+ (Fig. 3).

**Fig. 4:**
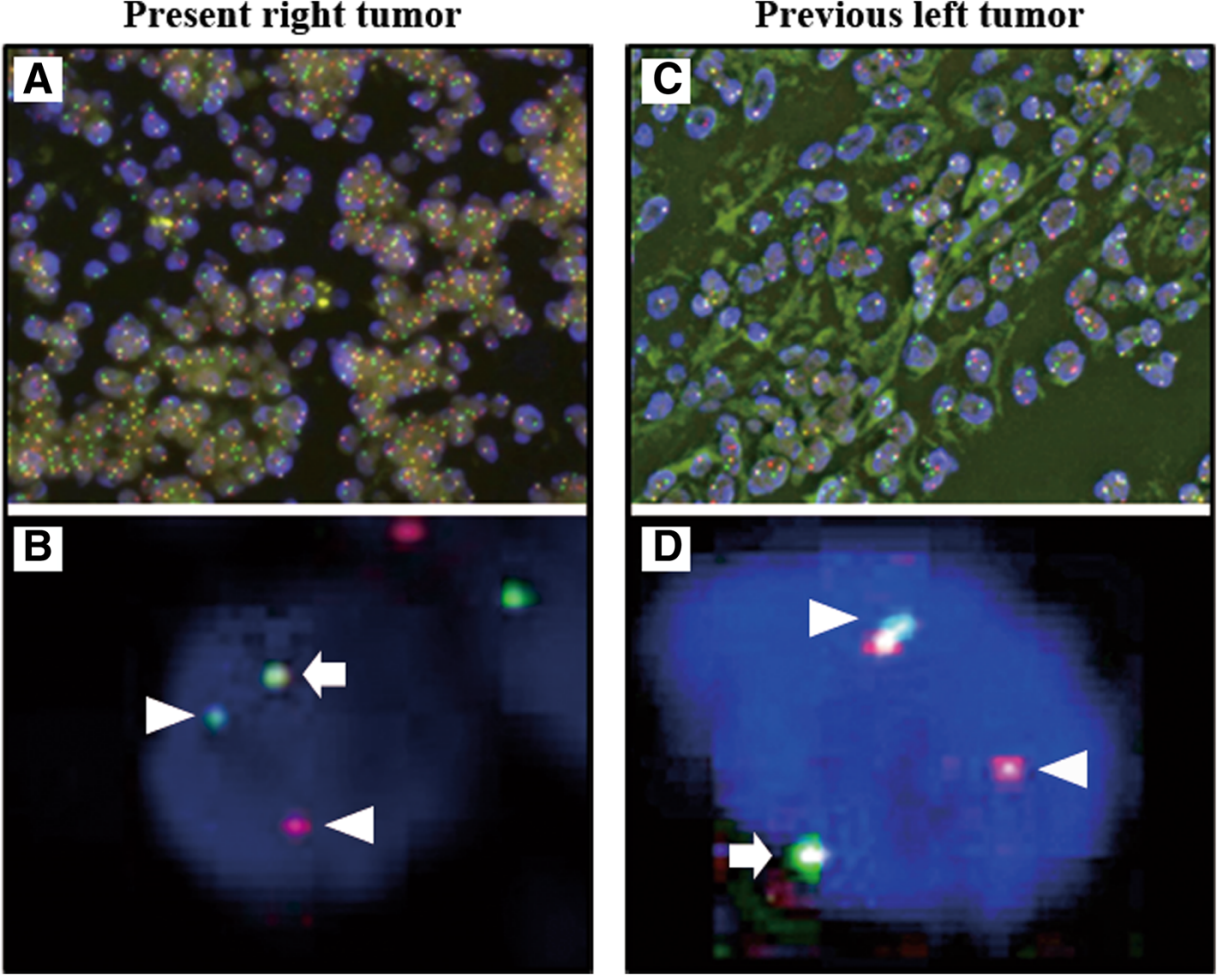
FISH analysis of TFE3 gene splitting of the present (**a** and **b**) and previous (**c** and **d**) tumor cells. A pair of split signals of TFE3 genes are shown as red and blue fusion fluorescence at high magnification (white arrow head). A green signal shows fused normal fluorescence of red and blue (white arrow)

We performed a dual-color, break-apart fluorescence in situ hybridization (FISH) assay to identify the chromosomal break point of TFE3 in paraffin-embedded tissue [8]. Briefly, the break-apart FISH assay with probes upstream and downstream to TFE3 showed red and green signals. A fused or closely approximated green-red signal pattern was interpreted as a normal result, whereas a TFE3 fusion resulted in a split-signal pattern. Signals were considered to be split when the green and red signals were separated by a distance of more than 2 signal diameters. For each tumor, a minimum of 100 tumor cell nuclei were examined under fluorescence microscopy at × 1000 magnification. Only nonoverlapping tumor nuclei were evaluated. Positive findings were defined as more than 10% of the tumor nuclei showing the split-signal pattern [9]. The TFE3 gene showed gene splitting in 71.55% of 130 neoplastic cells and in 76.82% of 233 neoplastic cells in the present and the previous tumor, respectively. Typical TFE3 break-apart signals of the present and previous tumors are presented in Fig. 4.

**Fig. 5:**
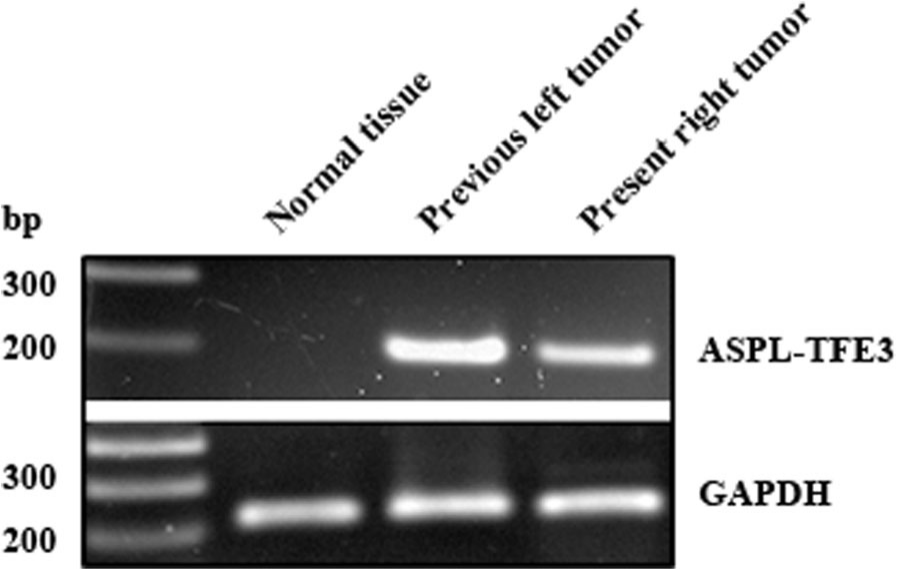
RT-PCR of ASPL-TFE3 fusion genes of the previous and present tumor tissue. Previous and present tumor expressed ASPL-TFE3 fusion gene, but not normal kidney tissue of the present. GAPDH expression of each tissue was confirmed as housekeeping gene

Total RNA was extracted from formalin fixed paraffin embedded tissue of the previous tumor and from frozen tissue of the present tumor using a standard organic extraction method (MACHEREY-NAGEL, Germany and QIAGEN, Germany, respectively). ASPL-TFE3 fusion transcripts were detected using an ASPL forward primer: 5’-AAAGAAGTCCAAGTCGGGCCA-3′ and a TFE3 exon 4 reverse primer: 5’-CGTTTGATGTTGGGCAGCTCA-3′. Glyceraldehyde-3-phosphate dehydrogenase (GAPDH) transcripts were detected using the forward: 5’-CGGATTTGGTCGTATTGG-3′ and reverse: 5’-TCCTGGAAGATGGTGATG-3’ GAPDH primers [2]. The ASPL-TFE3 fusion gene was detected in the tissue from the present and the previous tumor but was not detected in the normal tissue. GAPDH that was used as a loading control was detected in each reaction (Fig. 5).

There is a no evidence of recurrence at 8 months postoperatively.

## Discussion and conclusions

Xp.11.2 translocation RCC is a rare variety of RCC that was first described in 1995 by Dijkhuizen et al [10]. It is categorized as a separate entity in the 2004 World Health Organization classification of tumors of the urinary system [11]. This type of RCC frequently affects children and adolescents. Our patient was diagnosed as Xp11.2 translocation RCC at the ages of 49 and 56 years-of age. Patients of middle age and over with Xp11.2 translocation RCC have rarely been reported [12]. There is variation in the histological features of Xp11.2 translocation RCC such as clear cell, papillary, alveolar, and nested. Seventy five percent of adult Xp11.2 translocation RCC is predominately the clear cell histological type, whereas most pediatric cases consist of papillary histological features [13]. In our present left tumor, clear cell features were the predominant type, followed by alveolar and papillary. Also, characteristic findings such as eosinophilic, voluminous and clear cytoplasm led to the diagnosis of adult Xp11.2 translocation RCC with ASPL-TFE3 fusion.

Positive immunostaining of TFE3 and negative staining of TFEB excluded 6p21 translocation RCC. The results of positive immunostaining of RCCMa and CD10, and negative staining of Cathepsin K, HMB45 or Melan A also led to a diagnosis of ASPL-TFE3 fusion. Most previous cases of Xp11.2 translocation RCC showed positive staining of RCCMa and CD10. Negative staining of Cathepsin K supported ASPL-TFE3 fusion, while tumors with PRCC-TFE3 fusion mostly display positive staining of Cathepsin K [14]. Melanin may be upregulated in Xp11.2 translocation RCC with PSF-TFE3 and CLTC-TFE3 [7, 15]. Melanosome and Melanin A staining have not been reported in Xp11.2 translocation RCC with ASPL-TFE3 and PRCC-TFE3 fusion.

Our case is the first report of bilateral Xp11.2 translocation RCC. The next step was to consider whether the present tumor was due to metastasis from the previous tumor. Microscopic findings of the previous tumor revealed very large tumor cells, a discohesive area and rhabdoid features meaning more dedifferentiation and aggressiveness compared with the present tumor. These data suggest that these tumors occurred metachronously, and that the present tumor was not due to metastasis of the previous tumor.

We demonstrated the presence of the ASPL-TFE3 fusion gene that is the most common chimeric fusion gene resulting from the chromosome translocation that is characteristic of ASPSCR1. By using RT-PCR we also demonstrated that the tumors were negative for the PRCC-TFE3, PSF-TFE3, CLTC-TFE3 or NonO-TFE3 fusion genes. Analysis of von Hippel Lindau tumor suppressor gene mutation by direct sequencing and multiplex ligation-dependent probe amplification methods also gave a negative result (data not shown) [16]. These data supported the final diagnosis of bilateral Xp11.2 translocation RCC with ASPL-TFE3 fusion.

In conclusion, we present a case that may be diagnosed as bilateral Xp11.2 translocation RCC metachronously occurring. Immunohistochemical, cytogenetic and molecular findings allows the differential diagnosis of kidney neoplasms such as Xp11.2 translocation RCC.

## Abbreviations

AMACAR: Alpha-Methylacyl-CoA Race
ASPSCR1: Alveolar soft part sarcoma critical region 1
CA9: Carbonic Anhydrase IX
CD: Cluster differentiation
CLTC: Clathrin heavy chain
CT: Computed tomography
FISH: Fluorescence in situ hybridization
GAPDH: Glyceraldehyde-3-phosphate dehydrogenase
MRI: Magnetic resonance imaging
PRCC: Papillary renal cell carcinoma
PSF: PTB-associated splicing factor
RCC: Renal cell carcinoma
RCCMa: Renal cell carcinoma maker
RT-PCR: Reverse transcription-polymerase chain reaction
TFE3: Transcription factor E3
TFEB: Transcription factor EB
